# The Effectiveness and Safety of Total Glucosides of Paeony in Primary Sjögren's Syndrome: A Systematic Review and Meta-Analysis

**DOI:** 10.3389/fphar.2019.00550

**Published:** 2019-05-24

**Authors:** Zhe Feng, Bi-qing Zhang, Ya-mei Zhu, Bei-bei Yu, Ling Fu, Ling-ling Zhou, Xue-ping Zhou, Yan Lu

**Affiliations:** ^1^The First Clinical Medical College, Nanjing University of Chinese Medicine, Nanjing, China; ^2^Department of Traditional Chinese Medicine, The Third Affiliated Hospital of Soochow University, The First People's Hospital of Changzhou, Changzhou, China; ^3^Department of Rheumatology, Nanjing Hospital of Chinese Medicine, The Third Affiliated Hospital of Nanjing University of Chinese Medicine, Nanjing, China; ^4^School of Foreign Languages, Nanjing University of Chinese Medicine, Nanjing, China; ^5^Jiangsu Provincial Key Laboratory of Pharmacology and Safety Evaluation of Material Medica, School of Pharmacy, Nanjing University of Chinese Medicine, Nanjing, China; ^6^Department of Rheumatology, Affiliated Hospital of Nanjing University of TCM, Jiangsu Province Hospital of TCM, Nanjing, China

**Keywords:** *the total glucosides of paeony*, primary Sjögren's syndrome, effectiveness, safety, meta-analysis

## Abstract

**Objective:** To assess the effectiveness and safety of *the total glucosides of paeony* (TGP) on the treatment of primary Sjögren's syndrome (pSS) by conducting a meta-analysis.

**Methods:** Eight databases were searched from their inception to December 10, 2018 for randomized controlled trials (RCTs). The Revman 5.3 software was used for this meta-analysis.

**Results:** Nine RCTs which included 770 participants were identified. Pooled results showed that significant difference in Schirmer's test (*P* < 0.00001) comparing TGP with placebo (PBO). However, the pooled results displayed significant differences in salivary flow rate, Schirmer's test, erythrocyte sedimentation rate (ESR), C-reactive protein (CRP), rheumatoid factor (RF), serum γ-globulin, immunoglobulin G (IgG), IgA, IgM, and effective rate (*P* ≤ 0.01) in the co-administration of TGP with immunosuppressant (IS) compared with IS alone. Subgroup analyses revealed both heterogeneities in ESR and serum γ-globulin were eliminated, showing combined intervention of TGP + IS being more advantageous than single usage of IS (*P* < 0.00001). However, the advantage varied among three subgroups and showed a gradual weakening over time. Furthermore, our results showed statistical significance in Schirmer's test (*P* = 0.0006), when hydroxychloroquine (HCQ) was jointly applied, but not in the case of combined TGP with methotrexate (MTX) (*P* = 0.41). For the safety analysis, the most common adverse events (AEs) were diarrhea or gastrointestinal discomfort, and no severe AEs were reported in TGP group. Meanwhile, six trials showed statistically insignificant differences between TGP + IS and IS in AEs (*P* = 0.76).

**Conclusions:** Improving the lacrimal gland secretion (Schirmer's test) is the prominent function of TGP compared with PBO. TGP + IS can improve the clinical symptoms, such as lacrimal and salivary gland secretion function (Schirmer's test, salivary flow rate), inflammatory indices (ESR, CRP, and RF) and immunoglobulins (γ-globulin, IgG, IgA, and IgM) on the basis of IS monotherapy. In addition, TGP has an acceptable safety profile and AEs were not increased when TGP combined with IS in pSS. Therefore, TGP can be considered to be a potentially valid and safe drug for the treatment of pSS in the clinic. In view of the limitations of the included trials, the potential beneficial effectiveness and safety of TGP need additional high-quality, multi-center, and large-scale RCTs to assess its use in pSS treatment.

## Introduction

Sjögren's syndrome (SS) is a chronic systemic autoimmune disease characterized by the lymphocytic infiltration of exocrine glands in pathology and manifested by glandular dysfunction and classic sicca symptoms of dry eyes and mouth in clinic (Sandhya et al., 2017). Generally, SS falls under two headings, namely primary Sjögren's syndrome (pSS) and secondary Sjögren's syndrome (sSS) (Nair and Singh, 2017). sSS often arises along with other autoimmune diseases, such as rheumatoid arthritis (RA) and systemic lupus erythematosus (SLE), and the accompanying disease of sSS is always the main determinant of treatment decisions which leads to difficulty in evaluating the treatment of SS, so in this study we focus on pSS which normally occurs alone (Holdgate and St.Clair, 2016; Saraux et al., 2016; Both et al., 2017; Perzynska-Mazan et al., 2018).

In terms of its mobility in different genders of pSS, women are ten times more likely to men and may occur at any age, with a mean onset age ranging from 40 to 50 s (Patel and Shahane, 2014; Paiva and Rocha, 2015). According to latest epidemiological data, the prevalence of pSS is about 0.29 to 0.77% in China (Chinese Rheumatology Association, 2010), and the incidence and prevalence rates global wide are 6.92 (95% CI 4.98 to 8.86) per 100,000 persons and 60.82 (95% CI 43.69 to 77.94) per 100,000 persons, respectively (Qin et al., 2015). Key symptoms such as glandular features, musculoskeletal pain, and fatigue can result in harsh physical limitations, immense psychological distress, and severe complications such as lymphoma, even greater financial burdens on patients' family in long term, which has been considerably affecting their quality of life, making it a heated research focus for medical specialists worldwide (Lackner et al., 2017; Ng et al., 2017; Stack et al., 2017; Alunno et al., 2018).

With its pathogenesis remaining unclear, more and more studies have been working on identifying disease-associated molecular clusters or biomarkers so to on one hand clarify the complex pathogenesis of pSS, and on the other hand, to provide reference for early diagnosis and prevention in clinic (Nishikawa et al., 2016).

To date, pSS treatment protocols in clinic are normally determined in line with overall evaluation of patients' symptoms and extraglandular manifestations. At present, immunosuppressants/ Disease-modifying Anti-rheumatic Drugs (DMARDs) are the main strategies used against extraglandular manifestations, but they did not highlight the improvement of gland function and exist risk of adverse events (Saraux et al., 2016; Stefanski et al., 2017); biologic agents remain controversial and demand more RCTs to prove the efficacy (Barone and Colafrancesco, 2016; Carsons et al., 2017), which leaving current clinical treatment for pSS being limited. Therefore, a safer and more effective therapeutic strategy need to be explored.

The total glucosides of paeony (TGP) is the active compound extracted from the roots of *Paeonia lactiflora* Pall, a traditional Chinese medicinal herb, which has been reported with analgesic, anti-inflammatory, immunomodulatory, and antioxidant functions (He and Dai, 2011; Luo et al., 2016, 2017). TGP has been approved by the China Food and Drug Administration to be applied treating RA in 1998, then it has been used for the treatment of SLE and SS (Zhang and Dai, 2012; Zhao et al., 2012; Li et al., 2013). In recent years, TGP has been increasingly used in treating pSS, and clinical and experiment studies conducted by previous researchers have demonstrated that TGP can relieve its typical symptoms, improve gland function, and have functions of affecting the Th1/Th2 cytokine balance, decreasing the expression levels of tumor necrosis factor-α (TNF-α), interferon-γ (IFN-γ), inerleukin-4 (IL-4), and reducing the pathological damage of submandibular glands by upregulating AQP-5 and its mRNA expression, which suggested TGP may represent a potential novel therapeutic agent for the treatment of pSS in this modern age (Wu et al., 2015, 2016; Zhou et al., 2016).

Up to date, only one systematic review regarding the effectiveness and safety of TGP in the treatment of pSS has been reported (Jin et al., 2017). However, this review only included articles until July 2016. Meanwhile, the effectiveness and safety of TGP used alone were absent from this review, and the further in-depth analysis of indicators with heterogeneity was lacking too. Therefore, we aim to update and re-systematically review the effectiveness and safety of TGP in the treatment of pSS with regard to the latest published articles.

## Methods

### Search Strategy

We searched PubMed (Medline), Embase, Cochrane Central Register of Controlled Trials (CENTRAL), EBSCOhost, China National Knowledge Infrastructure (CNKI), Wanfang Med Database, Chinese Biomedical (CBM) Database, and Chinese VIP Information Database from their inception to December 10, 2018. On the other hand, websites of Clinicaltrials.gov and Chinese Clinical Trial Registry were thoroughly searched to confirm the availability of relevant unpublished studies. The languages were restricted in English and Chinese. For the English electronic databases, the search strategy was set as follows: (paeon^*^ OR TGP) AND (Sjögren's syndrome OR SS OR Sjogrens Syndrome OR Syndrome, Sjogren's OR Sjogren Syndrome OR Sicca Syndrome OR Syndrome, Sicca). For the Chinese databases, free text terms were used, such as “baishao zonggan” or “pa fu lin” (which means total glucosides of paeony in Chinese) and “gan zao zong he zheng” (which means Sjögren's syndrome in Chinese).

### Procedures

The Preferred Reporting Item for Systematic Reviews and Meta-Analyses (PRISMA) Statement (Moher et al., 2009) was taken as the guideline in our study. Besides, the protocol of this review was registered in PROSPERO, registration number is: CRD42018107570.

### Selection Criteria

Studies were included if they met the following criteria: (1) only the RCTs related to the effects of TGP in pSS were included in this review. Trials published in the form of dissertations were also included as eligible studies. (2) individuals diagnosed with pSS according to the international classification in 2002 (Vitali et al., 2002). (3) patients who received oral TGP alone or TGP in combination with IS in the experimental group, IS alone, or PBO as controls for at least 2 months. (4) the outcome measurements consisted of EULAR Sjögren's Syndrome Disease Activity Index (ESSDAI), EULAR Sjögren's Syndrome Patient Reported Index (ESSPRI), salivary flow rate, Schirmer's test, ESR, CRP, RF, serum γ-globulin, IgG, IgM, IgA, effective rate, and AEs. We excluded the studies of Non-RCTs, self-control, conference abstracts, as well as patients not indicated for primary Sjögren's syndrome.

### Data Extraction and Risk of Bias Assessment

Two authors (Z.F. and Z.B.Q.) extracted the relevant data independently according to the predetermined selection criteria. The data included the following: study design, year of publication, participant characteristics, diagnostic criteria, methodology, intervention and control approaches, treatment duration, outcome measures and AEs. The Cochrane risk of bias tool (Higgins and Green, 2011) was used to assess the methodological quality of each included study by the two authors. It included the following items: random sequence generation (selection bias), allocation concealment (selection bias), blinding of participants and personnel (performance bias), blinding of outcome assessment (detection bias), incomplete outcome data (attrition bias), selective reporting (reporting bias), other bias. The risk of bias was evaluated through using “Low,” “Unclear” and “High.” When disagreements occurred, two authors discussed to resolve these issues. If disagreements persisted, then the third author (Z.L.L.) was consulted to make the final decisions.

### Statistical Analysis

The Revman 5.3 software was used for this meta-analysis. For dichotomous data, relative risks (OR) was used to express; For continuous data, the mean difference (MD) or standardized mean difference (SMD) and a 95% confidence internal (CI) were used. The I-squared and Chi-squared tests were used to assess statistical heterogeneity. A random-effect model was applied if *I*-squared > 50% or Chi-squared test *p* < 0.1; otherwise, a fixed-effect model was adopted. A significance level of 5% was employed for all statistical data. Subgroup analysis was carried out when heterogeneity exists. In addition, we conducted sensitivity analyses to evaluate the results.

## Results

### Characteristics of Included Studies

**Figure 1:**
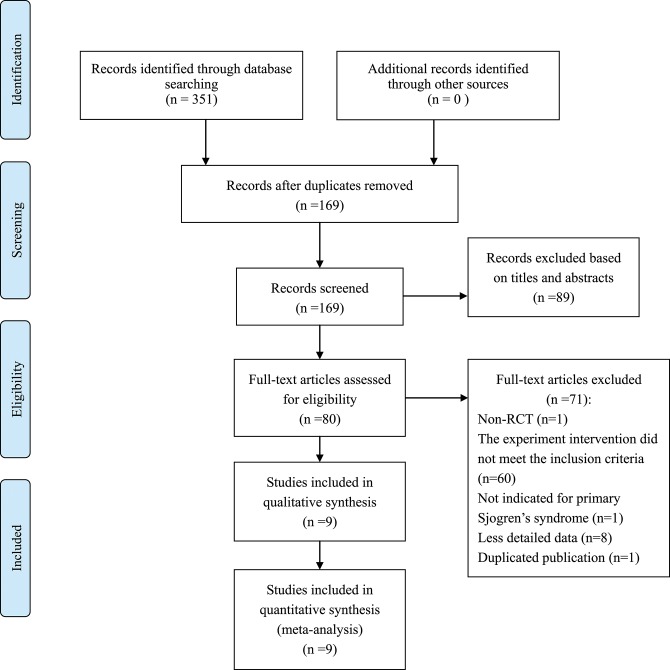
Flow chart of trials selection.

We identified 351 records and removed 182 for duplication reason. A total of 89 records were excluded after screening them based on the titles and abstracts. Our final analysis included 9 RCTs (Feng and Zhang, 2008; He, 2010; Cai, 2011; Yin, 2011; Zhao and Zhao, 2013; You and Wang, 2015; Liu and Deng, 2016; Zhou et al., 2016; Liu et al., 2018), with a total of 770 patients who were randomly grouped into TGP with PBO (*n* = 389) (You and Wang, 2015; Zhou et al., 2016; Liu et al., 2018), or co-intervention of TGP and IS (TGP + IS) with IS alone (*n* = 381) (Feng and Zhang, 2008; He, 2010; Cai, 2011; Yin, 2011; Zhao and Zhao, 2013; Liu and Deng, 2016) by further analyzing the remaining full-text articles. The flow chart of trials selection was shown in Figure 1. The characteristics of the included trials were presented in Table 1.

**Table 1:** The characteristics of the included trials.

**References**	**sex ration(M/F)**	**Sample size(M/F)**	**Age (year)**	**Intervention and dose**		**Duration (months)**	**Main outcomes**
		**Experimental Control**		**Experimental Control**			
Feng and Zhang, 2008	0/78	42 36	24–52	TGP 0.6 g tid+MTX 10 mg qw	MTX 10 mg qw	9 months	Salivary flow rate, Schirmer's test, ESR, serum γ-globulin, AEs
He, 2010	0/48	26 22	28–57	TGP 600 mg tid+HCQ 200 mg bid	HCQ 200 mg bid	3 months	Salivary flow rate, Schirmer's test, ESR, IgG, IgM, IgA, serum γ-globulin, AEs
Cai, 2011	7/53	30 30	22–64	TGP 0.6 g qd+MTX 10 mg qw	MTX 10 mg qw	6 months	Effective rate, salivary flow rate, Schirmer's test, RF, ESR, CRP, serum γ-globulin, AEs
Yin, 2011	0/81	42 39	35–61	TGP 600 mg tid+HCQ 200 mg bid	HCQ 200 mg bid	3 months	Salivary flow rate, Schirmer's test, RF, ESR, IgG, AEs
Zhao and Zhao, 2013	3/55	30 28	28–67	TGP 0.6 g tid+HCQ 100 mg bid	HCQ 100 mg bid	6 months	Salivary flow rate, Schirmer's test, ESR, IgG, IgM, IgA, AEs
Liu and Deng, 2016	8/48	28 28	24–55	TGP 0.6 mg, bid~tid+MTX 5 mg~10 mg, qw~biw	MTX 5 mg~10 mg, qw~biw	6 months	Effective rate, salivary flow rate, Schirmer's test, RF, ESR, CRP, serum γ-globulin, AEs
You and Wang, 2015	N/A	15 15	27–67	TGP 0.6 g, bid~0.6 g, tid	PBO	6 months	ESSDAI, ESSPRI, Stimulated salivary flow rate, Schirmer's test, AEs
Zhou et al. (2016)	6/39	29 16	N/A	TGP 0.6 g, tid	PBO	6 months	Stimulated salivary flow rate, ESR,AEs
Liu et al. (2018)	13/301	211 103	18-75	TGP 0.6 g, bid~0.6 g, tid	PBO	6 months	ESSPRI, Schirmer's test, Stimulated salivary flow rate, ESR, AEs

TGP, the total glucosides of paeony; Placebo, PBO; MTX, methotrexate; HCQ, hydroxychloroquine; ESR, erythrocyte sedimentation rate; CRP, C-reactive protein; RF, rheumatoid factor; immunoglobulin (IgG, IgM, and IgA); AEs, adverse events

### Quality of Included Studies

**Figure 2:**
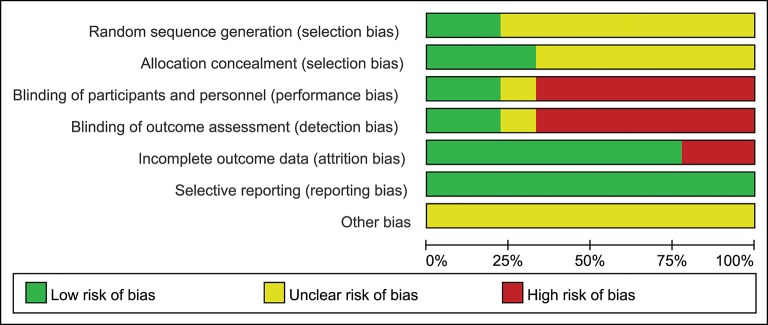
Risk of bias graph of included studies.

**Figure 3:**
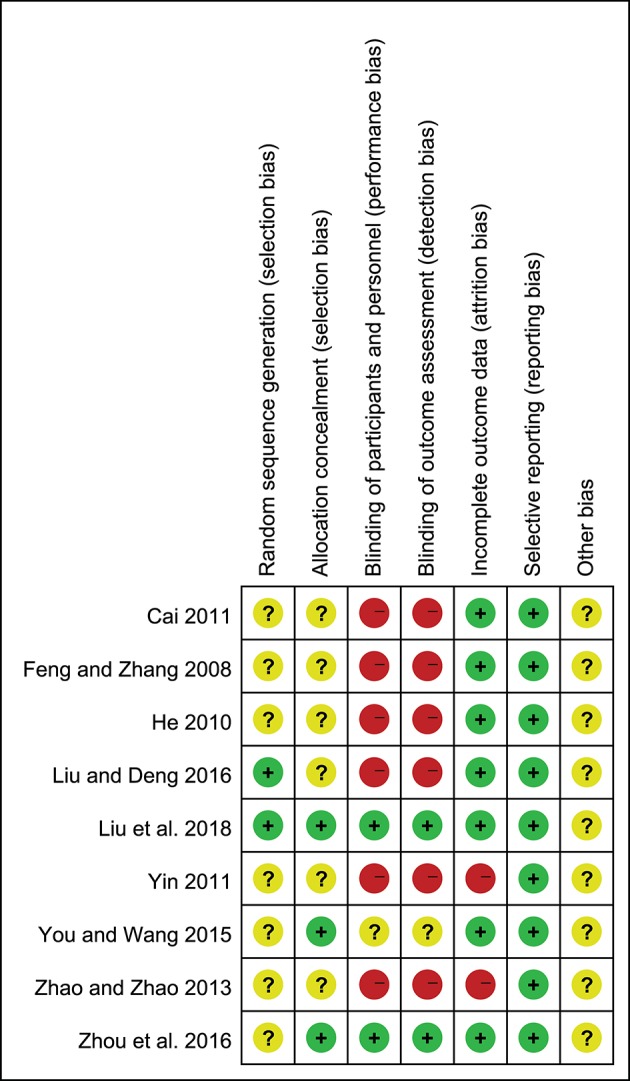
Risk of bias summary of included studies.

Only two of the nine trials (22.2%) reported the method of randomization, and the seven remaining trials mentioned randomization but did not describe the method in detail; three studies (33.3%) reported allocation concealment; two studies (22.2%) described the blinding of participants, personnel and outcome assessment; seven studies (77.8%) described the complete outcome data; all studies (100%) showed low risk of selective reporting and unclear risk of other bias (Figures 2, 3).

### Effects of Interventions

#### TGP vs. PBO

**Figure 4:**
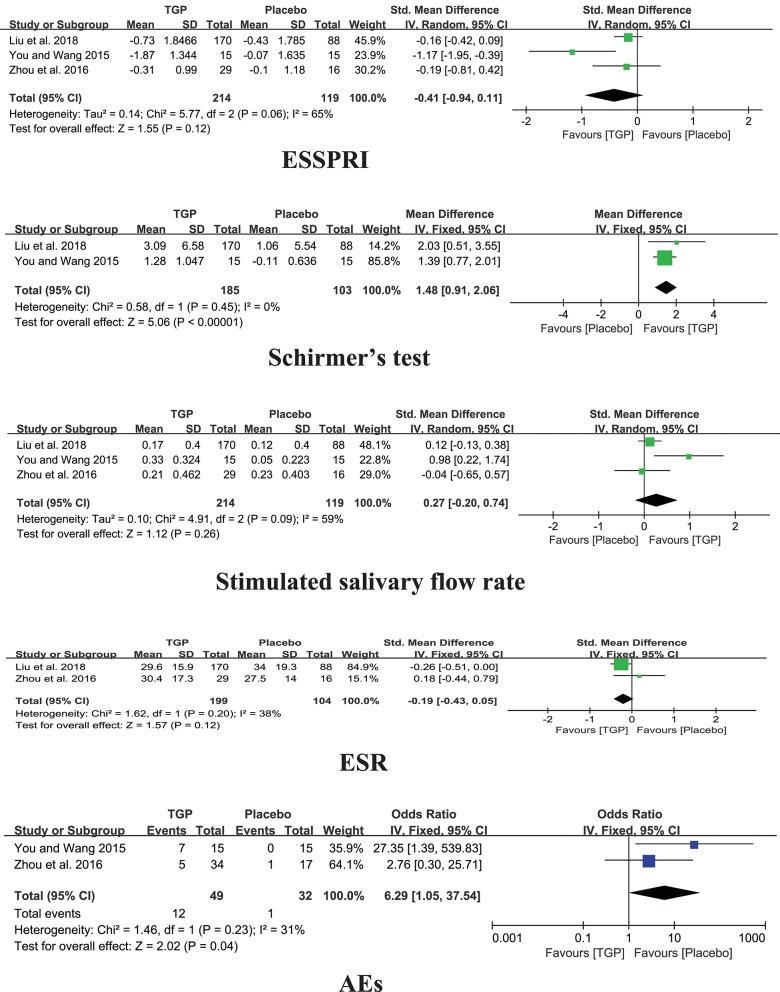
Forest plots of TGP compared with PBO.

Among the three studies comparing TGP with PBO, the pooled results showed significant difference in Schirmer's test (MD = 1.48, 95%CI: 0.91 to 2.06, *I*^2^ = 0%, *P* < 0.00001), aside from ESSPRI, stimulated salivary flow rate, and ESR (*P* > 0.05) (Figure 4).

#### TGP + IS vs. IS Alone

**Figure 5:**
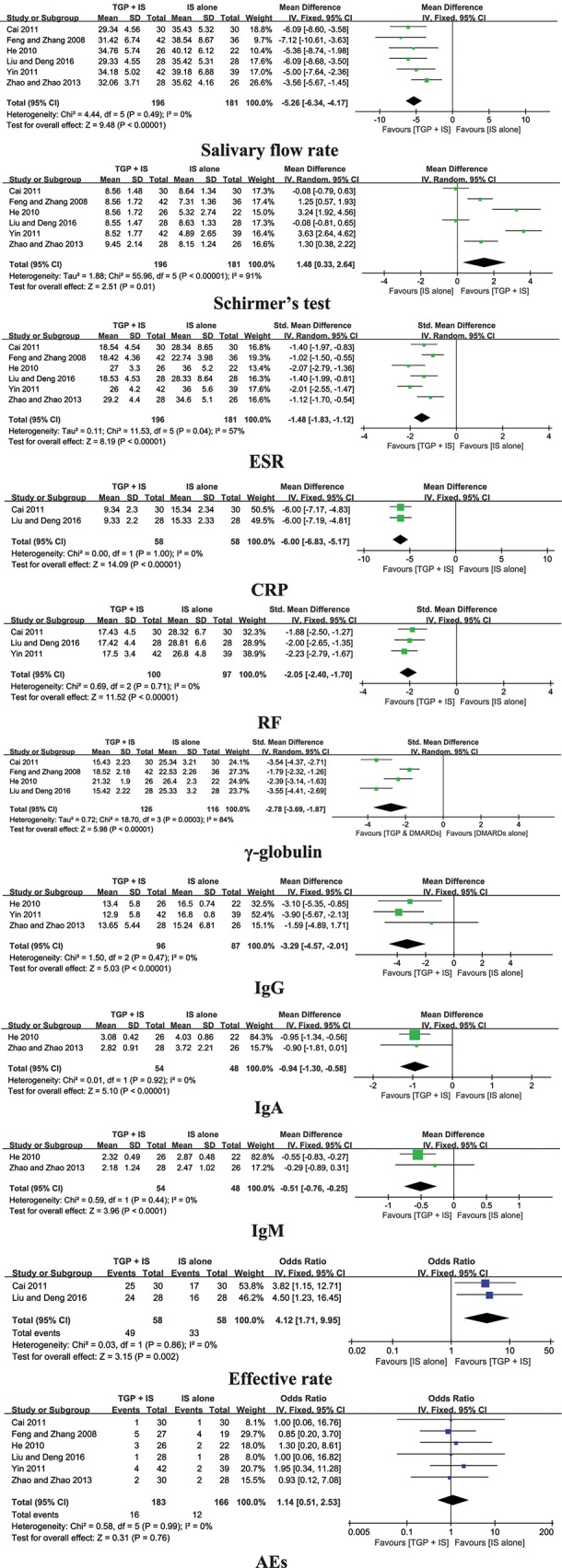
Forest plots comparing TGP+IS and IS.

Six trials compared TGP + IS with IS alone, and the pooled results displayed significant differences in salivary flow rate, Schirmer's test, ESR, CRP, RF, serum γ-globulin, IgG, IgA, IgM, and effective rate (*P* ≤ 0.01) (Figure 5).

Meanwhile, we conducted subgroup analyses based on different intervention duration due to high heterogeneity in Schirmer's test, ESR, and serum γ-globulin.

**Figure 6:**
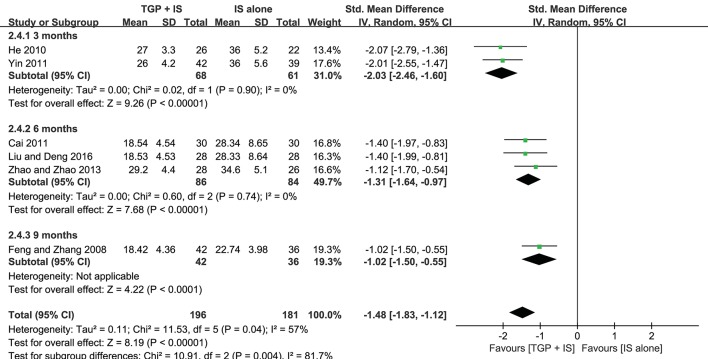
Subgroup analysis of ESR based on intervention duration of TGP + IS vs. IS alone.

**Figure 7:**
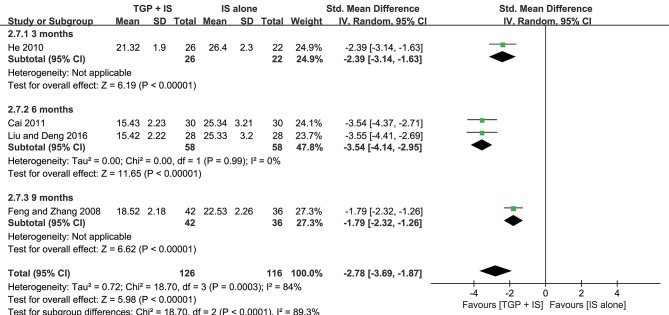
Subgroup analysis of serum γ-globulin based on intervention duration of TGP + IS vs. IS alone.

Both heterogeneities in ESR and serum γ-globulin were eliminated after subgroup analyses, and the results stayed consistent with the combined, showing combined intervention of TGP + IS being more advantageous than single usage of IS (*P* < 0.00001). However, the advantage varied among three subgroups and showed a gradual weakening over time, with 3 months (MD = −2.03, 95%CI: −2.46 to −1.60, *I*^2^ = 0%, *P* < 0.00001) significantly better than 9 months in ESR (MD = −1.02, 95%CI: −1.50 to −0.55, *P* < 0.0001), and with 6 months (MD = −3.54, 95%CI: −4.14 to −2.95, *I*^2^ = 0%, *P* < 0.00001) significantly better than 9 months in serum γ-globulin (MD = −1.79, 95%CI: −2.32 to −1.26, *P* < 0.0001) (Figures 6, 7).

**Figure 8:**
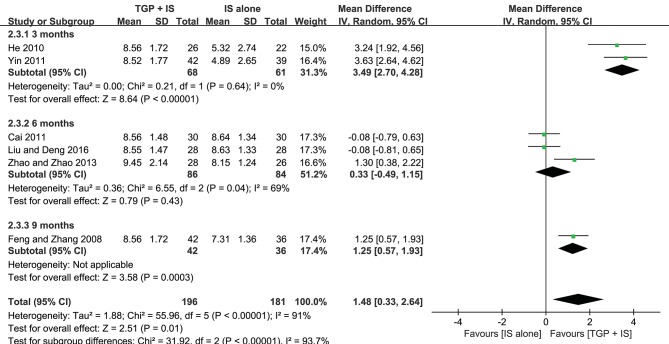
Subgroup analysis of Schirmer's test based on intervention duration of TGP + IS vs. IS alone.

**Figure 9:**
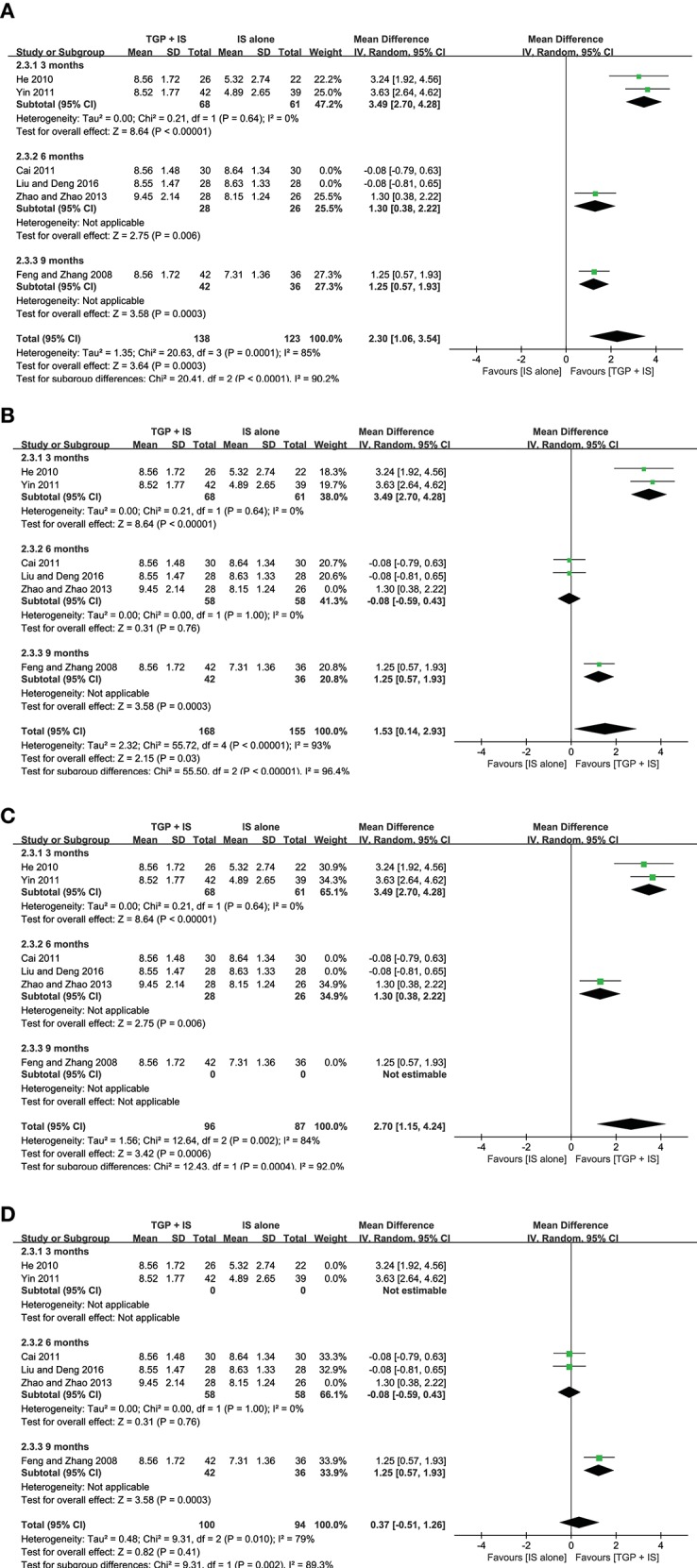
**(A–D)** Schirmer's test based on different intervention time (sensitivity analysis).

In view of the heterogeneity of Schirmer's test in 6 months still being high (*I*^2^ = 69%) (Figure 8), we further conducted sensitivity analyses and found out its heterogeneity may be mainly due to combined use with different IS during that intervention time, which explained removing the study about HCQ (Zhao and Zhao, 2013), the subgroup heterogeneity disappeared (*I*^2^ = 0) and removing the two studies using MTX (Cai, 2011; Liu and Deng, 2016) also eliminated the problem of heterogeneity. Also, we found that the 3-month and 9-month studies (Feng and Zhang, 2008; He, 2010; Yin, 2011) were taken HCQ and MTX as controls, respectively. Therefore, we conducted comparisons between the groups according to specific IS used. For HCQ, combined use of TGP with HCQ was advantageous than single its use, with its effect being significantly better in 3 months (MD = 3.49, 95% CI: 2.70 to 4.28, *I*^2^ = 0%, *P* < 0.00001) than in 6 months (MD = 1.30, 95% CI: 0.38 to 2.22, *P* = 0.006) (Figure 9A); as for MTX, combined use with MTX for 9 months is better than single use of MTX (MD = 1.25, 95% CI: 0.57 to 1.93, *P* = 0.0003), and significantly advantageous than 6 months, whose effect was even not better than using MTX alone (MD = −0.08, 95% CI: −0.59 to 0.43, *I*^2^ = 0%, *P* = 0.76) (Figure 9B), suggesting that when TGP was used in combination with different IS, there were differences in time effect accordingly.

**Figure 10:**
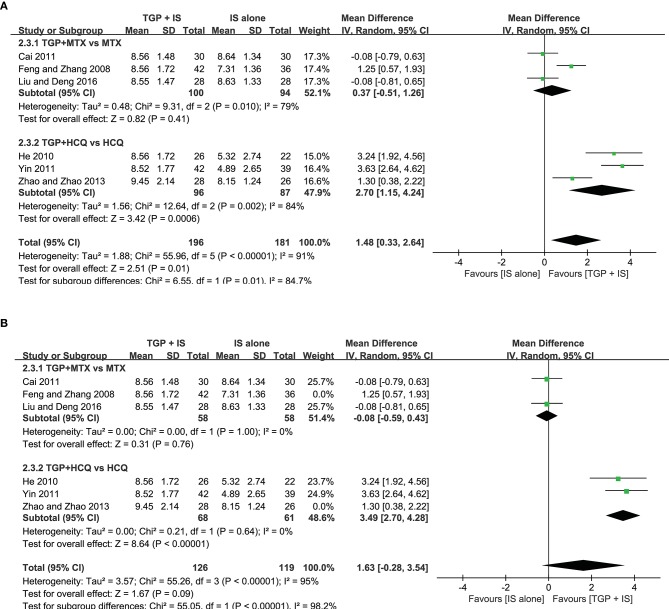
**(A,B)** Subgroup analysis and sensitivity analysis of Schirmer's test based on different IS use.

Furthermore, we re-analyzed each subgroup to explore possible differences resulted from the combined applications of different IS and discovered statistical significance was observed in Schirmer's test (MD = 2.70, 95%CI: 1.15 to 4.24, *I*^2^ = 84%, *P* = 0.0006) (Figure 9C), when HCQ was jointly applied, but not in the case of combined TGP with MTX (MD = 0.37, 95%CI: −0.51 to 1.62, *I*^2^ = 79%, *P* = 0.41) (Figure 9D). In view of the high heterogeneity of the two subgroups, further sensitivity analysis was performed and the relevant individual studies were excluded (Feng and Zhang, 2008; Zhao and Zhao, 2013), heterogeneities of the two subgroups were then eliminated and still have the effects, with significant difference between the two subgroups (MD = 3.49, 95% CI: 2.70 to 4.28, *I*^2^ = 0%, *P* < 0.00001; MD = −0.08, 95% CI: −0.59 to −1.43, *I*^2^ = 0 %, *P* = 0.76) (Figure 10).

### Safety of Interventions

#### TGP vs. PBO

Three included trials reported Adverse Events (AEs) (You and Wang, 2015; Zhou et al., 2016; Liu et al., 2018). Compared TGP with Placebo, there was a significant difference in AEs (*P* = 0.04), which reported mild to moderate diarrhea in nineteen patients, gastrointestinal discomfort in two patients, mild dizziness in two patients, and leukopenia in one patient in the TGP group which involved 260 patients totally. Moreover, no severe AEs were observed in the three trials.

#### TGP + IS vs. IS Alone

AEs were reported in all six trials (Feng and Zhang, 2008; He, 2010; Cai, 2011; Yin, 2011; Zhao and Zhao, 2013; Liu and Deng, 2016). Comparisons of AEs in TGP + IS group and IS alone group demonstrated insignificant differences (*P* = 0.76). The main AEs were stated as diarrhea, transaminase abnormality, and blurred vision in a few of participants who received TGP in combination with IS. Meanwhile, symptoms such as skin rash, blurred vision, and transaminase abnormality occurred in the groups who received IS alone. Two trials (Feng and Zhang, 2008; Zhao and Zhao, 2013) separately reported that one patient withdrew from the study due to severe diarrhea in TGP + IS group.

## Discussion

The main constituents of TGP, containing paeoniflorin, paeonin, albiflorin, hydroxy-paeoniflorin, and benzoylpaeoniflorin, have been reported to have anti-inflammatory and immunoregulatory activities (Wang et al., 2015). Previous studies showed that TGP plays a definite role in pSS treatment, reducing ESR and improving the symptoms of dry mouth and eyes (Li et al., 2006). In Jin's meta analysis that published in 2017, they compared efficacy differences resulted from co-administration of TGP and an immunosuppressant (IS) with the same IS alone, and found out the efficacy of “TGP+IS” was superior in terms of improving exocrine function (saliva flow test and Schirmer test), reducing inflammatory indices (ESR and CRP) and immunoglobulins (IgM and IgG). Meanwhile, AEs were found both similarly few in two groups. However, they did not analyze the efficacy and safety of TGP when it used alone; neither did they conduct further in-depth analysis of indicators with heterogeneity.

In our study, we included nine trials which contained TGP vs. PBO and TGP+IS vs. IS, along with a recent multi-center trial with high quality (Liu et al., 2018). Meanwhile, the heterogeneity was further processed through subgroup analysis and sensitivity analysis, which made our study, a more comprehensive version than Jin's. Comparing effects from single use of TGP and PBO, both beneficial effect in Schirmer's test and differences in AEs were observed in the two groups. When co-intervention of TGP and IS was applied, our results were in favor of Jin's, however indicate more beneficial effects in terms of regulating RF, serum γ-globulin and IgA levels, and increasing effective rate than using IS alone. Moreover, these beneficial effects may vary in accordance with intervention duration and different IS applied.

### Effect on Exocrine Gland Secretion Function

Our meta-analysis demonstrated that TGP could significantly improve Schirmer's test result compared to PBO. Meanwhile, the co-intervention of TGP with MTX or HCQ was superior to the single use of MTX or HCQ. It is suggested that TGP may have unique advantage in improving lacrimal gland secretion function, and can exert this effect advantage during its combined use.

However, we found that the combined use of different IS and intervention duration to be the determining factors responsible for the high heterogeneity in Schirmer's test through subgroup analysis and sensitivity analysis. Our meta-analysis demonstrated that co-intervention of TGP with HCQ could improve Schirmer's test result significantly compared with single usage of HCQ, but this advantage can be weakened with time; on the contrary, the co-intervention of TGP with MTX showed no significant difference compared with using MTX alone, but it can become better than MTX over time. As the current studies have found no clear effects of HCQ and MTX on Schirmer's test (Skopouli et al., 1996; Wang et al., 2017), the context mentioned above presented that co-intervening with HCQ may be more conducive to TGP to improve lacrimal gland secretion or synergistic reaction may take place when TGP and HCQ were used together, while in combination with MTX may not be conducive or have antagonistic action, and both the potential synergistic reaction and antagonistic action mentioned above may decrease over time. We also have found that there was published trial (Zhao and Zhao, 2013) confirming the observed more beneficial effect on Schirmer's test of TGP+HCQ than TGP (*P* < 0.01), which supports our inference that HCQ may result in synergistic reaction on Schirmer's test. However, we have not found any relevant literature reports on MTX antagonism, and there is still no evidence of the intervention time effect of HCQ or MTX, which requires further studies in the foreseeable future.

Meanwhile, our meta-analysis demonstrated that TGP had no statistical difference in the improvement of salivary flow rate compared to PBO, while the co-administration of TGP and the above IS was superior to single-use of IS. For salivary gland secretion, there was a study shown that HCQ can increase salivary flow rate in pSS patients (Mumcu et al., 2013); nevertheless, other study suggested that this effect was not obvious (Gottenberg et al., 2014). The recent published review showed very limited effect of HCQ in improving the salivary flow rate, but slightly higher function in the treatment of dry mouth than placebo group (Wang et al., 2017). The previous literature did not show the role of MTX in improving the salivary flow rate and changing objective parameters of dry mouth (Skopouli et al., 1996). The above suggests that TGP may have a weak effect on the improvement of salivary flow rate, and can enhance the effect of HCQ on increasing salivary flow rate when used with HCQ together, or promote the effect itself when co-intervention with HCQ or MTX.

### Effect on Inflammatory Indice

Our pooled data indicated that although TGP was not superior in decreasing ESR compared with PBO, ESR was improved significantly when TGP was combined with MTX or HCQ. According to the meta-analysis published by Wang et al. (2017) which showed that HCQ could reduce ESR compared with PBO, and some studies that revealed that MTX has anti-inflammatory activity and can improve inflammatory makers (Cipriani et al., 2014; Nair et al., 2017), we infer that TGP maybe have a synergistic effect of enhancing the anti-inflammatory activity of MTX or HCQ, and promoting their effects on improving ESR. However, the subgroup analysis suggested that the intensity of the synergistic effect of TGP may also diminish over time.

CRP and RF were significantly improved when co-intervention of TGP with MTX or HCQ compared with MTX or HCQ monotherapy, which further confirm that the combination of TGP with IS has a synergistic effect on improving the inflammatory markers.

### Effect on Immunoglobulins

All of the included three studies compared TGP with PBO referred to IgG and two referred to IgA and IgM. As no specific data given, it's unavailable to merge, but these studies had shown that TGP cannot reduce the immunoglobulins significantly. However, when TGP was combined with IS, the pooled data in our meta-analysis indicated that serum γ-globulin and three kinds of immunoglobulins (IgG, IgA, and IgM) were improved significantly. Previous studies have shown that HCQ can significantly reduce immunoglobulins in pSS patients (Fox et al., 1988), and MTX is also thought to have the role in reducing immunoglobulins. This suggested that TGP may have the synergistic effect of enhancing the immunomodulatory activity of MTX or HCQ, and reducing immunoglobulins. Meanwhile, we can also conclude that this intensity of synergistic effect on TGP has the possibility to decrease with time through the subgroup analysis.

### Effect on Disease Index

It had been pointed out that ESSDAI and ESSPRI now can be used as endpoints in therapeutical studies (Witte, 2018), which were the indicators for evaluating disease activity and symptom perception, respectively. In our three included trials of TGP compared with PBO, only one trial had ESSDAI score and was unable to merge, while there was no significant difference in ESSPRI. The included six trials of TGP+IS compared with IS did not mention about ESSDAI and ESSPRI. Although the pooled data from two trials of TGP+IS compared with IS suggested the advantage of TGP combination in effective rate, they only calculated the rate of the patients with the improvement degree ≥30% in the six indicators of the three aspects mentioned above. The calculation method needs further study and demonstrate. Therefore, in the follow-up study, it is necessary to involve ESSDAI and ESSPRI, and more comprehensive and complete clinical trials are needed to design and evaluate.

### Safety Assessment

Our review also displayed a more detailed safety assessment than the previous analysis. Although there was a significant difference in AEs compared TGP with Placebo, the most common AEs of TGP were diarrhea or gastrointestinal discomfort, and no severe AEs were observed. Moreover, we noticed that there's one article mentioning comparing TGP with IS alone, the result of which indicated an overall equivalent efficacy between TGP and IS with no obvious hepatotoxicity or ocular toxicity (Zhao and Zhao, 2013). This suggested that the main AEs of TGP were mild gastrointestinal reactions, and its safety may be superior to IS in comparison.

Comparing TGP+IS with IS alone, there were insignificant differences in AEs (*P*>0.05). In addition to the common AEs like transaminase abnormality and blurred vision when IS used alone, diarrhea is still the manifestation. However, AEs in some of patients were gradually relieved on the completion of the experiment without treatment in these included trials. This means that we can use TGP+IS to treat pSS in clinic without increasing safety risk.

The doses of TGP used in the nine trials varied 600 to 1,800 mg/day, effectiveness and safety in dose dependent or independent need more in-depth analysis if there are more high-quality trials in future. In view of this, more multi-center and large-scale RCTs are strongly needed for a more comprehensive and objective evaluation of the effectiveness and safety of TGP in patients with pSS in the foreseeable future.

### Quality Assessment

In this review, there are some limitations which we need to pay attention. Firstly, only nine trials met the inclusion criteria, and all these trials were implemented in China. This limitation causes out inability to precisely evaluate the role of TGP in patients with pSS. Secondly, the quality assessment of the methodology exhibited defects: six trials (66.7%) ran a high risk of bias in blinding of participants, personnel and outcome assessment, and two trials (22.2%) presented a high risk of bias in incomplete outcome data. Seven studies (77.8%) only mentioned randomization without referring to their specific methods, and six articles (66.7%) did not report the allocation concealment method. Thirdly, the random effect model was used in several outcome analyses due to high heterogeneity, which may be related to different doses and intervention duration of TGP and the combined use of different drugs. Fourthly, although eleven outcome measurements were chosen in “TGP+IS” vs. IS alone, some indexes, such as ESSDAI and ESSPRI were not mentioned, and some like CRP, IgA, and IgM, were only reported in two articles. Besides, six trials reported the effective rate, they were disregarded in the standard outcome measurement because of different benchmarks, and only two trials can pool the results. Fifthly, the included trials of 3–9 months cannot provide long-term outcome reports, we haven't known whether to prolong the time would observe AEs more clearly. These all should be taken into consideration when interpreting the results. Hence, the trial designs of high-quality, large-scale RCTs are strongly needed to evaluate the effectiveness and safety of TGP on patients with pSS.

## Conclusion

Our findings suggest that improving the lacrimal gland secretion (Schirmer's test) is the prominent function of TGP compared with PBO. The co-administration of TGP and IS would significantly improve the clinical symptoms of patients with pSS, such as lacrimal and salivary gland secretion function (Schirmer's test, salivary flow rate), inflammatory indices (ESR, CRP and RF) and immunoglobulins (γ-globulin, IgG, IgA, and IgM) on the basis of IS monotherapy. However, the effects may vary according to different IS and action time. Through the subgroup analysis, we found that co-intervening with HCQ may be more conducive to TGP to improve lacrimal gland secretion or synergistic reaction may take place when TGP and HCQ were used together, while in combination with MTX may not be conducive or have antagonistic action. The potential synergistic reaction and antagonistic action mentioned above may decrease over time. In addition, the advantages of improving ESR and serum γ-globulin also may diminish over time potentially. Although TGP had a higher incidence in AEs compared with Placebo, the most common AEs were diarrhea or gastrointestinal discomfort, and no severe AEs were observed. In comparison, its safety may be superior to IS. Furthermore, AEs were not increased when comparing TGP+IS with IS. Therefore, TGP can be considered to be a potentially valid and safe drug for the treatment of pSS in the clinic, and can be chosen to improve the lacrimal gland secretion or enhance the effect of IS for treating pSS. However, this review has some limitations that should be considered. Therefore, more multi-center and large-scale RCTs are strongly needed for a more comprehensive and objective evaluation of the effectiveness and safety of TGP in patients with pSS in the foreseeable future.

## Author Contributions

ZF designed this study and wrote the paper. ZF, BZ, and LF performed the literature database search, data collection, and data extraction. YZ, LZ, and YL performed data analysis and rationalization of the results. BY polished the written English. XZ gave advice in preparing the writing.

### Conflict of Interest Statement

The authors declare that the research was conducted in the absence of any commercial or financial relationships that could be construed as a potential conflict of interest.
